# Cardiac Rehabilitation for Heart Failure With Preserved Ejection Fraction: A Narrative Review of the Benefits and Challenges

**DOI:** 10.7759/cureus.98933

**Published:** 2025-12-10

**Authors:** Meshal Faleh Alenezi, Mohammed Ahmad Altawili, Aseel Saleh M Aladwani, Mohammed Ibrahim A Al Shaikh, Adel Abdulrahman M Alghamdi, Ziyad Farouq Mesfer Alghamdi, Rayan Tawfiq M Alghamdi, Raneem Abdulaziz A Al Luhaybi, Bilal Hafizullah Abdulhameed, Manar Mohamed Ibrahim Gabr Badawi

**Affiliations:** 1 Internal Medicine: Adult Cardiology, King Salman Armed Forces Hospital, Tabuk, SAU; 2 General Surgery, King Faisal Specialist Hospital and Research Centre, Tabuk, SAU; 3 Nursing, Princess Nourah Bint Abdulrahman University, Riyadh, SAU; 4 Internal Medicine, College of Medicine, Al Baha University, Al Baha, SAU; 5 Internal Medicine, Al Rayan Medical College, Madina, SAU; 6 General Practice, Saudi German Hospital, Madinah, SAU; 7 General Practice, Saudi German Hospital, Riyadh, SAU

**Keywords:** cardiac rehabilitation, challenges, heart failure with preserved ejection fraction, hfpef, quality of life

## Abstract

Heart failure with preserved ejection fraction (HFpEF) is an increasingly global health problem with high morbidity and mortality, complicated by heterogeneous pathophysiology and widespread comorbidities. Although pharmacotherapy has some benefits, non-pharmacological approaches, particularly cardiac rehabilitation (CR), play a crucial role in managing cardiovascular disease.

This narrative review critically evaluates the therapeutic potential of CR in HFpEF, focusing on its impact on functional capacity, diastolic function, health-related quality of life, and the challenges associated with its implementation. We synthesize current understanding of HFpEF pathophysiology and exercise intolerance, detail the components of modern CR programs, and discuss exercise training modalities, including aerobic, resistance, and varied-intensity protocols. Evidence indicates that CR, particularly supervised exercise training, consistently improves peak oxygen uptake, functional capacity, and quality of life, with a strong safety profile. However, its effects on diastolic function are less consistent. Significant barriers to CR uptake and adherence remain.

Future research should prioritize large, well-designed trials to optimize CR protocols, tailor interventions to specific HFpEF phenotypes, and develop practical strategies to overcome implementation barriers. Clinically, broader integration of comprehensive CR into standard HFpEF care is recommended, supported by policy measures to improve access and resource availability for these essential programs.

## Introduction and background

Heart failure with preserved ejection fraction (HFpEF) is defined by a left ventricular ejection fraction (LVEF) of ≥50%, along with evidence of structural or functional cardiac abnormalities. HFpEF has emerged as a predominant clinical challenge in cardiovascular medicine, characterized by heterogeneous pathophysiological mechanisms of cardiac dysfunction, systemic inflammation, metabolic dysregulation, and diverse clinical manifestations. As global demographics shift toward older populations, the prevalence of this condition has risen substantially, now constituting over 50% of all heart failure patients [1]. Despite its epidemiological significance, HFpEF continues to present considerable therapeutic challenges, with persistently high rates of hospitalizations and mortality that underscore the urgent need for improved management strategies [2,3]. However, recent pharmacological advances, such as GLP-1 receptor agonists like semaglutide, show beneficial effects primarily in patients with HFpEF and concomitant obesity. These findings have not yet been translated into guideline-level recommendations. In accordance with current ESC 2023 guidelines, SGLT2 inhibitors remain the only therapy with a Class I recommendation for HFpEF [4,5].

The clinical complexity of HFpEF is further heightened by its frequent association with multiple comorbid conditions, which contribute to substantial diagnostic and therapeutic challenges. Consequently, effective management requires integrated, multidimensional treatment strategies that address both cardiac and extracardiac pathophysiological processes [6,7].

Emerging evidence highlights the complementary role of non-pharmacological modalities, particularly structured exercise training (ET), as a cornerstone of HFpEF management. Beyond its cardioprotective effects, targeted physical rehabilitation has shown promise in ameliorating exercise intolerance, restoring skeletal muscle efficiency, and enhancing patient-reported quality of life (QoL) metrics. Exercise intolerance is characterized by reduced peak oxygen uptake (VO₂ peak) and impaired functional capacity during physical activity [8,9].

The integration of non-pharmacological approaches aligns with contemporary perspectives advocating for multimodal interventions in HFpEF care, where synergistic approaches may counteract the disease’s systemic nature. Structured ET, a central component of cardiac rehabilitation (CR), forms an integral part of this multimodal strategy [8,9]. CR is a structured, supervised, and multidisciplinary program that extends beyond conventional exercise by integrating individualized aerobic and resistance training, patient education, risk factor management, and psychosocial support. Unlike standard exercise programs, CR is tailored to optimize functional capacity, quality of life, and overall cardiovascular outcomes in patients with HFpEF [8,9].

The British Association for Cardiovascular Prevention and Rehabilitation (BACPR) defines CR as a coordinated set of interventions aimed at positively influencing the underlying causes of cardiovascular disease. This approach seeks to optimize patients' physical, mental, and social well-being, enabling them to maintain or resume optimal functioning within their communities. Through improved health behaviors, CR also aims to decelerate or reverse disease progression [10].

Despite growing interest in CR for HFpEF, several important gaps remain. Current evidence is limited by small sample sizes, heterogeneous patient populations, and variability in exercise protocols, making it difficult to establish standardized, phenotype-specific CR recommendations. Data on long-term outcomes, including mortality and hospitalization, are sparse, and practical barriers to program implementation, such as accessibility, insurance coverage, and patient adherence, are not well addressed. Addressing these gaps is essential to optimize CR interventions and inform broader integration into routine HFpEF care [11].

Therefore, this review systematically evaluates the therapeutic role of CR in HFpEF, focusing on its effects on functional capacity, diastolic function, and QoL. It also addresses implementation challenges, including healthcare system limitations and patient-specific barriers related to multimorbidity.

## Review

Methods

This review was conducted as a narrative synthesis aimed at summarizing and contextualizing the current evidence on cardiac rehabilitation and related interventions in patients with HFpEF. No meta-analysis or quantitative pooling of outcomes was performed due to heterogeneity in study designs, interventions, and reported endpoints across the included literature. As such, we did not calculate summary effect estimates. Instead, we qualitatively describe and compare findings across studies and present key quantitative results (including effect estimates, P-values, and confidence intervals) as reported by the original authors.

In April 2025, a targeted literature search was conducted in major biomedical databases, including PubMed, Scopus, and Google Scholar, to identify studies relevant to HFpEF diagnosis, management, and rehabilitation strategies. Search terms included combinations of the following keywords: “HFpEF,” “heart failure with preserved ejection fraction,” “cardiac rehabilitation,” “exercise training,” “diastolic dysfunction,” and “management.” Additional articles were identified by reviewing reference lists of key publications to ensure that important studies were not overlooked. Boolean operators were applied to refine results. Key references were also identified from the bibliographies of relevant studies. We did not apply any filters or limitations during the search process, except for focusing on English-language studies on human subjects published between 2015 and 2025.

The selection of studies was guided by relevant inclusion and exclusion criteria. We prioritized peer-reviewed clinical trials, observational studies, and guideline documents that provided insights into diagnostic modalities, therapeutic strategies, and patient outcomes in HFpEF. Studies focusing exclusively on heart failure with reduced ejection fraction (HFrEF) or unrelated cardiac conditions were excluded. Duplicate publications and non-English articles were not considered.

Pathophysiology of HFpEF and exercise intolerance

HFpEF is characterized by impaired ventricular relaxation, increased chamber stiffness, and elevated filling pressures, all of which limit the heart’s ability to augment cardiac output during physical activity. In addition to these central cardiac abnormalities, HFpEF is increasingly recognized as a systemic disorder involving endothelial dysfunction, reduced peripheral oxygen extraction, skeletal muscle abnormalities, chronotropic incompetence, and impaired ventricular-arterial coupling. These mechanisms collectively contribute to pronounced exercise intolerance, one of the defining clinical features of HFpEF. As a result, patients experience reduced peak oxygen uptake (VO₂peak), early onset of fatigue and dyspnea, and diminished ability to perform daily activities. Understanding these multifactorial contributors is essential for designing targeted exercise interventions capable of improving functional capacity in this heterogeneous patient population [12]. Details of the pathophysiology of HFpEF are presented in Table 1.

**Table 1:** Pathophysiology of heart failure with preserved ejection fraction (EFpEF) Abbreviations: HFpEF: Heart Failure With Preserved Ejection Fraction, Lv: Left Ventricle, BNP: B-type (or Brain) Natriuretic Peptide, Nt-Pro BNP: N-terminal Pro-B-Type Natriuretic Peptide, E/E' Ratios: Ratio of Early Mitral Inflow Velocity (E) to Early Diastolic Mitral Annular Velocity (E'), Vo₂: Oxygen Uptake (Volume of Oxygen Consumed per Minute), A-vO₂Diff: Arteriovenous Oxygen Difference

Category	Details
Pathophysiology	HFpEF is a complex systemic syndrome characterized by severe exercise intolerance despite preserved cardiac function at rest. Reduced peak oxygen uptake (VO₂peak) results from interactions between cardiovascular, peripheral vascular, and skeletal muscle defects. VO₂ is determined by the Fick principle (VO₂ = cardiac output × a-vO₂Diff); impairments in either component limit exercise capacity [12].
Clinical Presentation	Typical symptoms include exertional dyspnea, fatigue, and reduced exercise tolerance. VO₂peak is commonly decreased.
Diagnostic Criteria	HFpEF diagnosis requires typical heart failure symptoms and/or signs, preserved LVEF (≥50%), objective evidence of diastolic dysfunction or structural heart disease, and evidence of elevated filling pressures.
Diagnostic Modalities	Echocardiography assesses left atrial enlargement, LV wall thickness, E/e’ ratios, diastolic filling patterns, and pulmonary artery systolic pressure; Biomarkers: elevated BNP or NT-proBNP supports increased cardiac filling pressures; Functional assessments: Cardiopulmonary exercise testing, stress echocardiography, or invasive hemodynamic measurement if noninvasive findings are inconclusive [12].
Purpose	These criteria and diagnostic modalities ensure accurate classification of HFpEF and alignment with contemporary guideline recommendations.

Cardiac and vascular contributions to exercise limitation

Chronotropic incompetence, characterized by an impaired ability to appropriately increase heart rate during exercise, represents one of the important cardiac mechanisms that can contribute to reduced VO₂ peak in HFpEF, although it is not universally present among all patients. Despite relatively preserved stroke volume, the failure to adequately elevate cardiac output because of impaired heart rate responses greatly restricts oxygen delivery with exercise [13-15]. In addition, exercise-induced rises in LV filling pressures resulting from compromised relaxation and impaired ventricular stiffness may lead to pulmonary congestion and exertional dyspnea, thereby increasing fatigue perception [16-18].

Beyond the heart itself, endothelial dysfunction and arterial stiffness are primary factors in reducing exercise capacity in HFpEF. Normally, endothelial cells modulate vasodilation in proportion to increased shear stress and metabolic demand. In HFpEF, this response is attenuated. Reduced flow-mediated dilation and increased arterial stiffness, particularly in the central arteries such as the thoracic aorta and carotids, result in elevated afterload, which inhibits LV-arterial coupling and further cripples cardiac function under stress [19,20]. Also, microvascular dysfunction of the skeletal muscle suppresses reactive hyperemia and redistribution of blood flow, reducing oxygen delivery to the exercising tissue [21,22].

Skeletal muscle and peripheral factors

Abnormalities in skeletal muscle are also a major predictor of decreased exercise tolerance in HFpEF. Both structural and metabolic changes play a role. Decreased muscle mass, especially in the lower limbs, and adipose tissue infiltration in muscle compartments are present in these patients [23]. These changes not only decrease oxygen extraction capacity but also impair oxidative metabolism and mitochondrial function. Histological studies have shown a tendency away from oxidative (type I) fibers and toward more glycolytic (type II) fibers, and that is correlated with decreased endurance [24]. At the same time, mitochondrial content and function deficits, such as diminished activity of citrate synthase and interrupted mitochondrial fusion, interfere with cellular yield of energy and further restrict aerobic performance [25].

Components of cardiac rehabilitation (CR) in HFpEF

**Figure 1:**
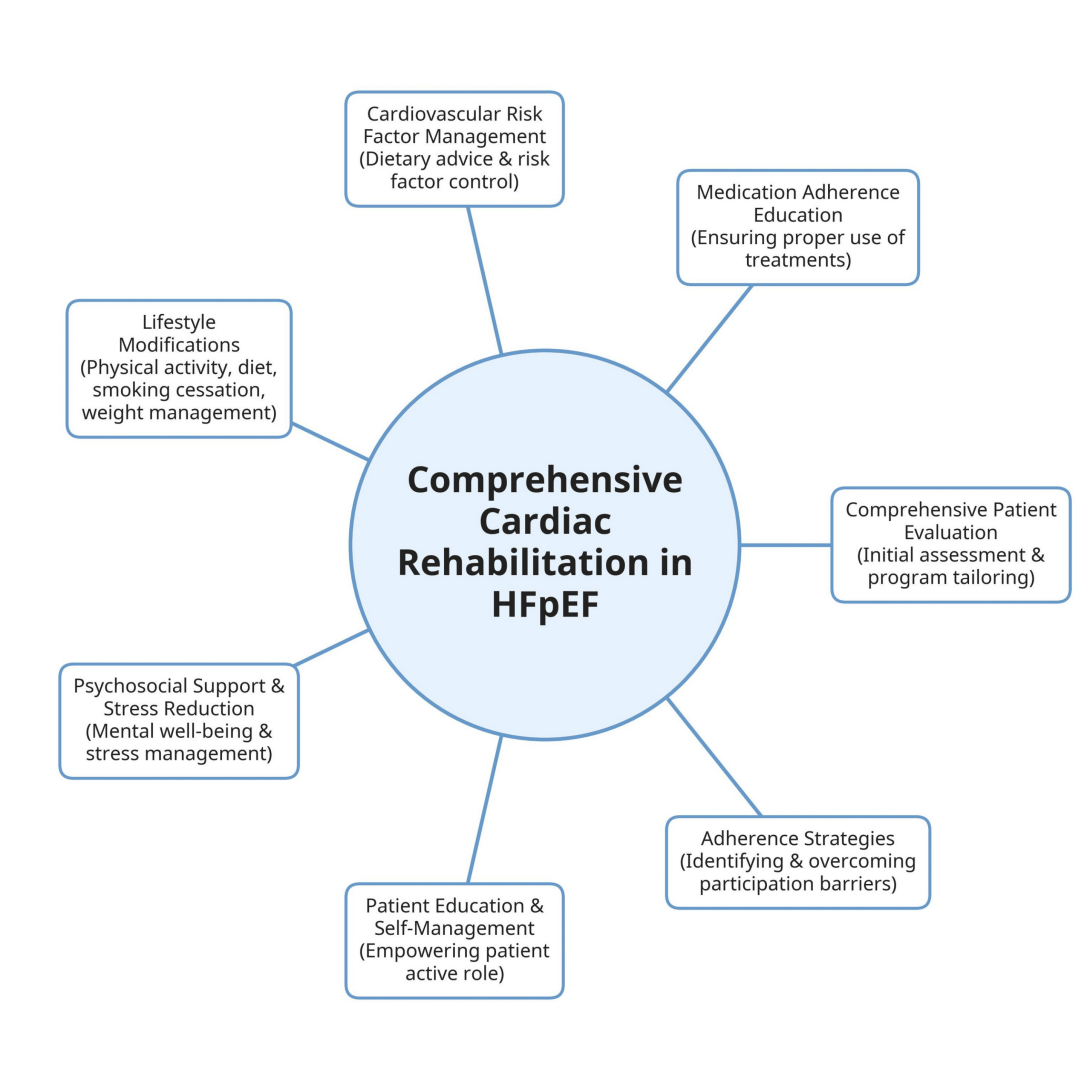
Core components of comprehensive cardiac rehabilitation (CR) for patients with heart failure with preserved ejection fraction (HFpEF) The figure illustrates the central role of CR in HFpEF management, highlighting key programmatic elements. Image credits: The author

Cardiac rehabilitation, a multidisciplinary strategy, aims to boost cardiovascular health. Contemporary CR for heart failure has developed into a comprehensive treatment that goes well beyond ET under supervision (Figure 1). For instance, many essential elements are incorporated into modern programs, such as comprehensive patient evaluation, medication adherence education, and cardiovascular risk factor management (including customized dietary recommendations). Also, lifestyle modifications, support for cessation of smoking, stress reduction methods, and the identification of possible obstacles to treatment adherence are among the elements included in the CR program. CR has evolved over the last 30 years from a simple, monitored exercise program to a thorough, interdisciplinary therapy approach backed by strong clinical data [26-28].

The structure of CR teams may vary across institutions, but they typically include core members such as a physician medical director (often a cardiologist), specialized nurses, advanced practice providers, exercise physiologists, and registered dietitians [29]. These professionals preferably work in close collaboration with referring physicians, patients, and their families, aiming to improve expected outcomes. Many programs also incorporate behavioral health specialists and clinical pharmacists as either integral team members or on a consultative basis when specialized interventions are needed. Notably, in patients with cardiovascular disease, some of the positive effects of CR in lowering cardiovascular mortality and hospitalizations have been linked to cholesterol levels, blood pressure, and a decrease in smoking, together with exercise [30,31]. This emphasizes that the main goals of CR are not only to enhance physical health and QoL but also to help individuals with heart failure acquire the skills they need to effectively self-manage [32]. Consequently, some elements that maximize cardiovascular risk reduction, encourage healthy habits and compliance, lessen disability, and encourage an active lifestyle for patients with heart failure and cardiovascular disease should be included in a CR program [28].

Programs that only include ET are not regarded as CR programs. Multidisciplinary comprehensive programs that incorporate self-care techniques beyond ET have been demonstrated in randomized controlled trials to considerably boost exercise capacity and lower hospitalization and mortality rates [33]. Davidson et al. reported that 79%of the usual care group was alive compared with 93% of people in the intervention group (P=0.03) at the 12-month follow-up [33]. A lower lifetime risk for heart failure and better preservation of cardiac structure and function are linked to lifestyle modifications that include physical activity, avoiding obesity, quitting smoking, eating a balanced diet, lowering cholesterol, and normalizing blood pressure and glucose levels [34]. Folsom et al. reported that 25.5% of participants developed heart failure by the age of 85. This lifetime heart failure risk was 14.4% for those with a middle-aged Life’s Simple 7 score of 10-14 (optimal), 26.8% for a score of 5-9 (average), and 48.6% for a score of 0-4 (inadequate). Among those with no clinical cardiovascular event, the prevalence of left ventricular hypertrophy in late life was approximately 40%, and diastolic dysfunction was approximately 60%, among those with an optimal middle-age Life’s Simple 7 score, compared with an inadequate score [34].

Exercise training modalities

*Aerobic Vs. Resistance Training* 

Aerobic interval training (AIT) is a structured exercise approach in which bouts of high-intensity aerobic activity are interspersed with periods of lower-intensity recovery. This modality is designed to enhance cardiovascular fitness, improve oxygen uptake (VO₂peak), and induce favorable cardiac, vascular, and skeletal muscle adaptations more efficiently than continuous moderate-intensity exercise. AIT is frequently implemented in both healthy individuals and patients with cardiovascular disease, including heart failure, to improve functional capacity while maintaining exercise tolerance. Evidence suggests that AIT can enhance exercise performance more effectively than conventional endurance training in heart failure populations [35-37]. In a key trial, Wisløff et al. demonstrated that AIT produced a 46% increase in VO₂peak compared with 14% with moderate continuous training (P=0.001), accompanied by reverse LV remodeling. Specifically, LV end-diastolic and end-systolic volumes decreased by 18% and 25%, respectively, LVEF increased by 35%, and pro-brain natriuretic peptide levels declined by 40%. Improvements in endothelial function and mitochondrial efficiency in the lateral vastus muscle were also observed exclusively in the AIT group [35]. Preclinical studies further support AIT, showing reductions in ventricular hypertrophy, improved contractile function, and decreased atrial natriuretic peptide expression in post-infarction heart failure models [38,39]. For individuals with multiple comorbidities, aerobic exercise training (ET) is widely recommended as a non-pharmacologic intervention to enhance functional capacity and overall health [40-42]. Details of key exercise training modalities, physiological effects, and clinical outcomes in HF are presented in Table 2.

**Table 2:** Summary of key exercise training modalities, physiological effects, and clinical outcomes in heart failure Abbreviations: AIT: Aerobic Interval Training, MCIT: Moderate-Continuous Intensity Training, VO₂peak: Peak Oxygen Consumption, HF: Heart Failure, LV: Left Ventricle, LVEF: Left Ventricular Ejection Fraction, BNP: B-type Natriuretic Peptide, ET: Exercise Training, QoL: Quality of Life, HIIT: High-Intensity Interval Training, HFpEF: Heart Failure with Preserved Ejection Fraction, HFrEF: Heart Failure with Reduced Ejection Fraction, RCT: Randomized Controlled Trial, LA: Left Atrium, E/e′: Ratio of Early Mitral Inflow to Mitral Annular Early Diastolic Velocity, Ex-DHF: Exercise Training in Diastolic Heart Failure

Topic	Key Findings	References
AIT vs. MCIT	AIT is superior to MCT in improving VO₂peak and cardiac remodeling in HF. Wisløff et al. demonstrated marked improvements in VO₂peak (46% vs. 14%), LV remodeling, LVEF (↑35%), BNP (↓40%), and endothelial and mitochondrial function.	[35]
Aerobic ET and Functional Capacity	Aerobic ET in sedentary individuals can increase VO₂peak by 10–20%, helping restore physiological function and improve QoL.	[43]
HIIT in HFpEF vs. HFrEF	HIIT/AIT improves VO₂peak and LV diastolic function in HFpEF (Angadi et al.) and shows stronger effects in HFrEF (Wisløff et al.). However, some studies in HFpEF show no improvement in LVEF.	[35,44]
Resistance + Aerobic Training in HFpEF	Combination training with calorie restriction improves body weight, physical function, QoL, muscle quality (+21%), and leg strength (+16%). Both aerobic-only and combined groups improve arterial stiffness and LV mass; neither prevents loss of lean mass during weight loss.	[45]
Long-term (12-month) Combined Endurance + Resistance Training in HFpEF	A large multicenter RCT found no improvement in modified Packer score (primary outcome), but significant clinical benefits: improved symptom class (×8 likelihood), increased VO₂peak, and good safety. No improvement in E/e′, consistent with most prior studies—except smaller trials (e.g., Ex-DHF pilot) showing diastolic benefits.	[46-48]
Reasons for Conflicting Diastolic Findings	Variability in HFpEF phenotypes, patient age, and severity of structural disease (e.g., larger LA volume; older age) may influence responsiveness to ET. Earlier-stage HFpEF (stage B) shows greater potential for improved myocardial stiffness with tailored ET.	[47,49]
Safety and Clinical Outcomes	No differences in hospitalization or mortality between exercise vs. usual care groups, confirming excellent safety and QoL improvements.	[47]
Combined Aerobic + Resistance Training in HFrEF	Shown to improve muscle strength, endurance, exercise duration, QoL, and sometimes cardiac function; some studies show similar benefits across modalities, with resistance training safely increasing exercise oxygen consumption without added cardiac risk.	[50-55]

*Moderate-Intensity Vs. High-Intensity Training* *(HIIT)*

The main clinical symptom of HFpEF is exercise intolerance that can be defined by decreased VO₂ peak [56]. ET has been identified as a suggested intervention to improve aerobic capacity and QoL in individuals with this condition. ET significantly increases VO₂peak, as meta-analyses have consistently demonstrated [57,58]. Current research indicates that these effects are mostly mediated by noncardiac processes. Subsequent multicenter trials in both HFpEF and HFrEF populations showed equivalent results between HIIT and MICT, despite earlier single-center studies suggesting higher efficacy of HIIT. HIIT involves repeated short bouts of vigorous aerobic exercise, typically performed at 80-95% of peak heart rate or VO₂ max, interspersed with periods of low-intensity recovery, with total session durations generally ranging from 20 to 40 minutes. In contrast, moderate-intensity training (MICT) consists of sustained aerobic exercise performed at 50-70% of peak heart rate or VO₂ max for 30-60 minutes per session, without intermittent rest periods [59,60].

The Ex-DHF pilot research, a randomized clinical trial that looked at supervised exercise in HFpEF, found that it improved QoL metrics and partially alleviated diastolic dysfunction [61]. The exact pathophysiological processes that underlie the positive effects of exercise on HFpEF are still not fully understood. Preemptive HIIT implementation protected skeletal muscle function in a hypertensive rodent model of HFpEF; however, neither HIIT nor MICT significantly improved skeletal muscle contractility in an obesity-related HFpEF model, according to preclinical research [62,63]. According to a previous study by Mueller et al., patients with HFpEF experienced similar outcomes in peak VO₂ with HIIT, MICT, or standard of care [59]. On the other hand, a study conducted by da Silveira et al. found that HIIT outperformed MICT in terms of increasing aerobic capacity [64].

Vascular dysfunction is a key pathophysiological feature of heart failure and other cardiovascular diseases. In individuals with HFpEF, this dysfunction typically affects all arterial segments and is linked to worse clinical outcomes [65,66]. Peripheral arterial tonometry measures microvascular endothelial dysfunction, flow-mediated dilatation indicates poor vasodilation in conduit arteries, and pulse wave analysis indicates increased arterial stiffness in large vessels [67,68]. Of note, single-center trials have demonstrated similar improvements in arterial stiffness or flow-mediated dilatation following MICT [58,65]. The consequences on HFpEF are more complex, even while ET consistently raises VO₂peak and enhances vascular function in patients with HFrEF. Improved peripheral oxygen extraction through improved vascular-skeletal muscle interaction and oxygen diffusion/utilization contributes to VO₂peak improvements in HFpEF [69].

The OptimEx-Clin trial, a multicenter study that randomly assigned 180 HFpEF subjects to either HIIT, MICT, or standard care, recently published its findings [68]. At the 3-month and 12-month follow-up periods, the trial's primary results showed no statistically significant difference in the peak VO₂ improvement between the HIIT and MICT groups. Both HIIT and MICT conferred similar improvements in aerobic capacity, with HIIT showing a mean increase of 1.5 mL/kg/min and MICT 2.0 mL/kg/min at three months; neither reached the predetermined clinically meaningful threshold of 2.5 mL/kg/min compared to controls, highlighting that regimen selection should be individualized based on patient characteristics and preferences [68].

Important clinical findings have been obtained from a recent meta-analysis of ET methods in HFpEF. In terms of enhancing cardiorespiratory fitness, HIIT did not seem to be more effective than moderate-intensity interval training (MIIT), which contradicts earlier beliefs on the advantages of higher-intensity exercise. Although HIIT has demonstrated considerable improvement in certain diastolic function measures, including the E/A ratio, it has no discernible effect on other important echocardiographic measurements, which downgrades its beneficial effect. These findings point to the lack of a definite benefit of HIIT over MIIT in improving systolic function or aerobic capacity in HFpEF patients [70]. Further research is necessary to investigate the plausible processes underlying exercise-induced benefits in this population.

Efficacy and benefits of CR in HFpEF

Effects on VO₂ Peak

Several randomized controlled trials (RCTs) and meta-analyses have shown in the literature that structured ET improves VO₂ peak in patients with HFpEF in a way that is clinically significant. In comparison to control groups, supervised ET programs improved VO₂ peak by an average of 2.2-2.8 mL·kg⁻¹·min⁻¹ (a 14% improvement), according to a prior meta-analysis that included 16 RCTs [71]. These results are considered in line with previous systematic reviews and meta-analyses that found increases of between 1 and 2.5 mL·kg⁻¹·min⁻¹ [72,73]. It is noteworthy that 1.0 mL·kg⁻¹·min⁻¹ (6-7%) is the clinically meaningful threshold for heart failure patients. In their analysis of nine studies, Leggio et al. discovered that ET greatly enhanced ventilatory threshold, peak VO2, and distance traveled in the 6MWT in individuals with HFpEF [74]. By examining eight trials, Fukuta et al. showed how aerobic exercise affected patients with HFpEF and found that exercise capacity significantly improved [72].

After a year of ET, Fujimoto et al. found that 11 HFpEF patients had a slight rise in their VO2 peak [75]. Particularly, ET was primarily responsible for a 16% improvement in peak arterial-venous oxygen variation [58]. In a group of 23 elderly individuals, Maldonado-Martin et al. also found that walking and cycling at 50% to 70% of VO₂peak intensity for 3 days a week for 16 weeks increased the VO2 peak [76]. In the same context, a previous meta-analysis of 8 RCTs revealed that structured exercise training (SET) raised baseline VO₂ peak in the intervention group from 15.8 to 18.0 mL·kg⁻¹·min⁻¹. However, controls had a minor decrease compared to their initial baseline (16.2 to 15.9 mL·kg⁻¹·min⁻¹). The robustness of exercise effects in this population was confirmed by the fact that these improvements persisted across trials that carefully defined HFpEF as ejection fraction (EF) ≥50% [77].

*Effect on 6-Minute Walk Test (6MWT)* 

Although the 6MWT is traditionally used to evaluate functional capacity in HFpEF, there is still inconsistency in its relationship to VO₂ peak. According to meta-analyses, ET increased 6MWT distance by 9%, estimated to be about 40 meters, which is deemed clinically significant [77]. Recent RCTs, however, have revealed that increases in 6MWT do not always correspond with modifications in VO₂ peak, indicating that these tests assess distinct physiological processes [76].

A previous RCT found no significant correlation between changes in 6MWT distance and VO₂ peak following ET [78]. This hypothesis probably challenges the assumption that the 6MWT serves as a reliable surrogate for cardiopulmonary exercise testing (CPET) in elderly HFpEF patients. Despite earlier results, a clinical investigation proposed that 6-MWT could be helpful in assessing functional status in women with HFpEF [79].

Moderate correlations between 6MWT and VO₂ peak have been recorded in previous HFrEF studies, suggesting that HFpEF and HFrEF may have different exercise physiologies [80]. The 6MWT is still useful for evaluating functional mobility in HFpEF in the real world, despite these differences. Several studies, especially those that involve walking-based interventions, have demonstrated that improvements in 6MWT are associated with improved symptom assessment and QoL, even with the lack of proportionate increases in VO₂ peak. This implies that rather than only measuring aerobic capacity, the 6MWT may more accurately represent both musculoskeletal and cardiovascular responses to exercise.

Effect on Mortality and Hospitalization

The evidence currently available on the impact of CR on hospitalization and mortality in patients with heart failure differs depending on the HF phenotype. For instance, CR did not significantly lower death or hospitalization rates for patients with HFrEF, according to pooled individual participant data from 18 RCTs with 3,912 participants [81]. Although these early investigations had some limitations, including smaller sample sizes, their results are consistent with other meta-analyses such as the ExTraMATCH research and a previous Cochrane review [82-84]. The large HF-ACTION experiment demonstrated that the neutral results were constant across all investigated subgroups, including age, sex, and illness severity [85].

However, according to observational evidence, HFpEF might have different outcomes. Participation in CR was linked to considerably better results in patients with HFpEF, according to a recent multicenter retrospective analysis with a median 2.4-year follow-up [86]. Composite outcomes (all-cause death and HF hospitalization) were 28% less likely to occur in patients undergoing CR, which shows a possible benefit of CR in these patients. For instance, there were notable decreases in all-cause mortality (33% lower risk) and HF hospitalizations (18% lower risk). These inconsistent findings across different HF phenotypes raise concern about the necessity for CR CR-modified approach according to each HF phenotype [86].

Effect on Quality of Life (QoL)

Exercise therapies have been shown to improve QoL in a number of studies. The authors of a meta-analysis that included individuals with HFpEF found that ET had no discernible positive effects on the mental or emotional aspects of QoL in these patients [72]. Nevertheless, the included trials did not consistently report QoL subscales. A meta-analysis of 3 RCTs with 115 HFpEF patients found insignificant positive effects of ET on depression in HFpEF patients, which is consistent with this finding [87]. To find out how ET affects HFpEF patients' mental or emotional states, larger RCTs are required.

Safiyari-Hafizi et al. found that patients who participated in a combined home-based interval and resistance training program experienced significant improvements in their QoL compared to conventional therapy [88]. Similar advantages were observed in aerobic training groups, which showed notable improvements in functional ability as determined by 6MWTs and several QoL areas (physical, psychological, social, and environmental) [89,90]. Meta-analyses demonstrating that physical activity enhances exercise capacity and QoL in patients with HFrEF corroborate these findings [91,92].

In patients with HFpEF, the association between physical function and health-related quality of life (HRQOL) is an important area of study. Although several studies have found moderate associations (r=0.30-0.58) between HRQOL measurements and baseline physical function, it is yet unknown how these outcomes alter dynamically after ET [72,93,94]. This is vital given that ET is presently the only intervention that has been shown to reliably improve the significantly reduced exercise capacity in HFpEF, with somewhat inconsistent effects on HRQOL. By identifying these associations, more focused interventions that address physical function and QoL outcomes in this patient population.

Challenges and limitations

Heart failure patients continue to underutilize CR despite explicit guidelines; throughout the past ten and a half years, participation rates in CR have been continuously below 20% in the United States and Europe, and frequently as low as 5% [95,96]. The significantly higher engagement observed in individuals with coronary heart disease after acute cardiac events contrasts sharply with this poor uptake in the case of HF [97]. Patients with prevalent comorbidities, including atrial fibrillation and chronic pulmonary disease, are often excluded from clinical trials included in available meta-analyses [72]. A majority of these trials used inconsistent diagnostic criteria that do not accord with current guidelines. These limitations make the implementation of CR for HFpEF particularly challenging and restrict the applicability of study results to actual HFpEF populations, which frequently exhibit several concomitant disorders [98,99].

Limited program availability and stringent insurance policies severely restrict access to cardiac rehabilitation at the healthcare system level, particularly for patients with HFpEF, who frequently do not meet CMS coverage requirements (LVEF ≤ 35% and NYHA class II-IV) [100]. Clinical difficulties, such as inadequate clinician knowledge of CR advantages and a lack of uniform training in referral processes, are exacerbated by these systemic hurdles. Patient-level challenges are equally significant; the average HF patient has several comorbidities, as about 80% of heart failure patients have at least three comorbidities. Physical deconditioning often falls into demographic groups that have historically been underrepresented in CR programs, including socioeconomically disadvantaged groups, elderly women, and ethnic minorities [101,102].

Clinical implications and practice recommendations

Managing HFpEF continues to present a substantial clinical challenge, as evidenced by the low success of randomized controlled studies in achieving primary endpoints. Given this treatment gap, non-pharmacological therapy gained more interest in research, particularly the role of physical activity in improving functional capacity. Although the best training regimens are still unknown, current research supports the use of structured exercise programs that combine resistance and aerobic training modalities. Current recommendations support moderate-intensity aerobic exercises under supervision (20-60 minutes, 3-5 sessions per week) using common equipment such as stationary bikes or treadmills, with progression based on tolerance levels.

It is necessary to have an integrated strategy that considers their nutritional status, mental health, and health literacy. Evaluating dietary protein and calorie consumption is also important, especially for people with muscle wasting, which can be hidden in obese patients. Baseline evaluations and recurring reassessments should include routine neuropsychological screening for mood disorders and cognitive impairment. In addition to therapy, group education provides rehabilitation techniques and validates patients' concerns. Patients should be better educated to make informed choices through the use of shared decision aids. Health care systems can invest in education and rehabilitation by following policies that include rehabilitation referrals as a publicly disclosed performance indicator and increasing the number of referrals.

Future directions

The landscape of CR for HFpEF is evolving, yet several critical areas demand further investigation to optimize patient outcomes and broaden accessibility. Future research should prioritize large-scale, multicenter, randomized controlled trials with robust methodologies to definitively establish the long-term benefits of CR on mortality, hospitalization rates, HRQoL, and cost-effectiveness in diverse HFpEF populations. These trials must incorporate more precise assessments, including detailed evaluations of right heart function, pulmonary circulation, and LV diastolic parameters, potentially exploring sex-specific responses to exercise [103,104].

A significant knowledge gap exists concerning the HFpEF with the pulmonary hypertension (HFpEF-PH) phenotype; dedicated studies are urgently needed to determine the safety and efficacy of tailored CR programs for this high-risk subgroup. Furthermore, the development and validation of personalized exercise prescriptions, considering individual patient characteristics such as comorbidities, frailty, and baseline exercise capacity, are paramount. This includes investigating the optimal FITT (frequency, intensity, time, type) principles for various HFpEF presentations and integrating CR with guideline-directed medical therapy (GDMT) more effectively [105].

Addressing the challenge of suboptimal CR uptake requires innovative approaches. Future trials should rigorously evaluate alternative delivery models such as home-based, mobile, and digitally assisted programs. These models hold promise for improving access and adherence, particularly for marginalized groups including the elderly, women, ethnic minorities, and socioeconomically disadvantaged individuals. Research into the integration of primary care into the long-term management and follow-up of CR participants is also warranted to enhance sustainability and adherence [106].

Finally, there is a pressing need to develop and assess affordable and sustainable CR models for low- and middle-income countries, ensuring equitable access to these vital services globally. Emphasis on patient-reported outcomes and implementation science research will be key to translating research findings into effective clinical practice [106].

Strengths and limitations of the review

The current evidence base supporting the management of HFpEF offers several strengths, including an expanding number of clinical trials and observational studies that provide increasingly detailed insights into pathophysiology and therapeutic responses. Furthermore, recent trials have contributed valuable data on the potential role of exercise, pharmacologic therapies, and multidisciplinary approaches, such as cardiac rehabilitation, in improving functional capacity and quality of life.

However, this evidence base also has important limitations. Considerable heterogeneity exists across studies in diagnostic definitions, inclusion criteria, and outcome measures, making cross-study comparison challenging. Many trials are limited by small sample sizes, short follow-up durations, or highly selected patient populations that may not fully reflect real-world HFpEF demographics. Additionally, there are gaps such as the limited long-term data and the potential role of tele-rehabilitation as an emerging care model. In addition, variability in the reporting of quantitative outcomes reduces interpretability. The scarcity of large, robust, randomized controlled trials evaluating comprehensive rehabilitation strategies further constrains the strength of recommendations. Together, these limitations highlight the need for more rigorous, standardized, and methodologically consistent research to strengthen the evidence base guiding HFpEF care. Importantly, the present work is a narrative review without a formal meta-analysis; therefore, the conclusions rely on qualitative synthesis rather than pooled effect estimates and should be interpreted accordingly.

## Conclusions

Cardiac rehabilitation, encompassing structured exercise training and comprehensive patient care, provides significant benefits for patients with HFpEF by improving functional capacity and quality of life. Healthcare providers should actively promote and refer eligible HFpEF patients to cardiac rehabilitation programs, recognizing it as an essential, evidence-based component of management. At the healthcare system level, policy development should focus on expanding the availability of cardiac rehabilitation services and improving financial coverage to enhance patient access and reduce disparities.

Future research should prioritize large-scale, well-designed trials to optimize cardiac rehabilitation protocols, determine the most effective exercise prescriptions for different HFpEF phenotypes, and evaluate the long-term impact on hard clinical endpoints such as mortality and hospitalization rates.
